# Factors associated with neonatal deaths in Chitwan district of Nepal

**DOI:** 10.1186/s13104-015-1807-3

**Published:** 2015-12-26

**Authors:** Rajani Shah, Bimala Sharma, Vishnu Khanal, Usha Kumari Pandey, Anu Vishwokarma, Dinesh Kumar Malla

**Affiliations:** Shree Medical and Technical College, Bharatpur, Chiwan Nepal; School of Public Health, Curtin University, Perth, Australia; Birendra Multiple Campus, Bharatpur, Chitwan Nepal

**Keywords:** Child health, Case–control survey, Nepal, Neonatal death

## Abstract

**Background:**

Neonatal mortality has remained unchanged since 2006 in Nepal. Reducing neonatal mortality is indispensable to reduce child mortality. The objective of this study was to investigate the factors associated with neonatal mortality. This study assesses socio-demographic factors, maternal health care and newborn care practices contributing to neonatal deaths in Chitwan district of Central Nepal.

**Methods:**

A case–control study was conducted during April–July 2012. The study used a mixed-method approach, in which records of neonatal deaths were obtained from the District Public Health Office and a comparison group, survivors, was obtained from the same community. A total of 198 mothers (of 99 neonatal deaths and 99 survivor neonates) were included in the survey. Focus group discussions, in-depth interviews and case studies were also conducted. Maternal characteristics were analyzed using descriptive statistics, Mc Nemar’s Chi square test and multivariable backward conditional logistic regression analysis. Qualitative data were analyzed by narrative analysis method.

**Results:**

More than four-fifth of mothers (86 %) had antenatal check-up (ANC) and the proportion of four or more ANC was 64 %. Similarly, the percentage of mothers having institutional delivery was 62 %, and postnatal check-up was received by 65 % of mothers. In multivariable analysis, low birth weight [adjusted odds ratio: 8.49, 95 % CI (3.21–22.47)], applying nothing on cord [adjusted odds ratio: 5.72, 95 % CI (1.01-32.30)], not wrapping of newborn [adjusted odds ratio: 9.54, 95 % CI (2.03–44.73)], and no schooling of mother [adjusted odds ratio: 2.09, 95 % CI (1.07–4.11)] were significantly associated with an increased likelihood of neonatal mortality after adjusting for other confounding variables. Qualitative findings suggested that bathing newborns after 24 h and wrapping in clean clothes were common newborn care practices. The mothers only attended postnatal care services if health problems appeared either in the mother or in the child.

**Conclusion:**

Results of this study suggest that the current community based newborn survival intervention should provide an even greater focus to essential newborn care practices, low birth weight newborns, and female education.

## Background

Globally, a total of 6.3 million children under-5 died in 2013; of which, about 42 % occurred during neonatal period [1]. The 2.8 million newborn children died mainly from preventable causes such as preterm birth complications (35.7 %), intrapartum complications (23.4 %), and sepsis (15.6 %) [2]. Pre-term birth is often characterized by low birth weight and is considered an important indirect cause of neonatal death [2]. Around three quarters of neonatal deaths occur within 6 days after birth [2]. Based on the progress made during the Millennium Development Goal (MDG) period (2000–2015) and the challenges posed by neonatal mortality, the World Health Organization (WHO) proposed new targets for reduction of neonatal mortality to below 12 per 1000 live births to be achieved by 2030 [3].

Three delay model explains the reasons behind such deaths that includes first delay in taking decision to seek care, second delay in arrival to a health facility, and third delay in receiving adequate care at health facility [4, 5]. Delay in decision to seek care is influenced by who makes the decision like individual, spouse, relatives or family, status of women, perceived severity of illness, distance to health facility, financial and opportunity cost, previous exposure to health care system and perceived quality of care. The second delay in reaching to health facility is affected by the availability of health facility, travel time, availability and cost of transportation and condition of roads. Similarly, third delay in receiving adequate care at the facility is influenced by referral system; shortages of supplies, equipment, and human resources; and skills of the available human resources [5].

The major direct causes of neonatal death in Nepal have been identified as infection, birth asphyxia/trauma, prematurity, and hypothermia [6]. Commonly associated causes of neonatal deaths in Nepal include poor pre-pregnancy health, inadequate care during pregnancy and during delivery, low birth weight and inadequate newborn and postpartum care [6].

The difficult terrain in Nepal has encouraged the national government to implement community-based service delivery approaches in improving maternal and child health across the country. Specifically to reduce neonatal mortality, in 2007 the Ministry of Health and Population of Nepal started a Community-Based Newborn Care Program (CB-NCP) [6]. This program relies on the Nepal’s extensive network of female community health volunteers (FCHVs), who are tasked with identifying newly pregnant women, encourage antenatal care attendance and institutional delivery, advise for or accompany the mother to the health facility during delivery, provide home-based newborn care visits on day one, three and seven, assess newborn and refer those that develop newborn complications to appropriate health facilities [7].

Such community-based interventions are believed to have contributed to reductions observed in the under-5-mortality rate since 2006 [8]. Progress in neonatal mortality, however, has not achieved the same level of success [8, 9]. Since 2006 the neonatal mortality rate (NMR) has remained stagnant at 33 deaths per 1000 live births [10]. Given this background, this study aimed to identify the socio-demographic factors, maternal and newborn health care practices that contributed to neonatal deaths in Chitwan district of Nepal.

## Methods

### Study setting

This study was conducted in Chitwan district of Central Nepal. The total population of district is 579,984. It had 36 Village Development Committees (VDCs) and 2 municipalities [11] at the time of data collection. Some of the VDCs have recently been merged to make new municipalities. The VDC and municipality are the lowest administrative units of rural and urban places for Nepal, respectively. In 2011 there were a total 14 birthing centres providing 24-h delivery services in the rural areas (VDCs) of the district [11]. The district has also one public hospital and two teaching hospitals providing tertiary level care. These are located in the urban areas (two municipalities) of the district. In addition, other private hospitals located in the urban area also provide health services. In 2010 CB-NCP was implemented in Chitwan district with the aim of reducing neonatal mortality and increase the recommended neonatal care practices [7, 12]. All health workers and FCHVs in the district were trained on standard newborn care. Core newborn care practices and all neonatal deaths reported according to reporting system of this program are likely to be an accurate method of reporting newborn mortality due to its community-based reporting system, which is tracked by health volunteers and health workers. Therefore, this study chose to use neonatal deaths occurring during a 1 year period in the district of Chitwan due to its accurate surveillance of neonatal deaths.

### Study design and participants

This was a case–control study conducted on the neonatal deaths that were retrieved from the District Public Health Office (DPHO) based on the surveillance system implemented through the CB-NCP. A comparison group, survivors, was selected in equal number from the same ward of the VDCs where the death had occurred. Structured interviews were carried out between April 2012 and July 2012 with mothers of the live births born during April 16, 2011 to April 15, 2012 who died during the neonatal period and mothers of the comparison group. For qualitative data, focus group discussions (FGDs), in-depth interviews and case studies were conducted between September and November, 2012.

### Data collection

List of neonatal deaths disaggregated by VDCs was collected from DPHO. Other factors such as ethnicity and sex of neonatal deaths were also identified from the same record. FCHVs were visited by trained enumerators at their homes. The FCHVs helped track the mother of deceased newborns. The mothers were then interviewed in their homes. For each neonatal death a ‘survivor’ of the neonatal period (and alive during the time of survey) was selected from the same VDC and ward to make comparison. Out of the record of 110 neonatal deaths, two cases were found to have been mistakenly reported by FCHV as neonatal deaths. Another five cases were found to be still births, one case had migrated to India with her husband, two were unreachable during the data collection period, and one case refused to take part in the interview. Therefore, a total of 99 neonatal deaths were included in this study with 99 ‘survivors’. A total of 198 mothers were interviewed using structured questionnaire. Pretesting of structured questionnaire was done in similar settings and eight enumerators were trained while pre-testing. The questionnaire included questions on socio-demographic characteristics and birth weight of the newborn; maternal health care services such as antenatal care (ANC), delivery care and post natal care (PNC); newborn care practices- instrument used for cord cutting, substance applied on the cord, drying, wrapping, bathing, breast feeding, and pre-lacteal feeding; health care seeking practices- distance to health facility, decision-making, first point of contact; and knowledge of danger signs.

Two FGDs were conducted with FCHVs, each with nine members. The aim of the FGDs was to identify community factors that may have contributed to the neonatal death. In addition, two case-studies were held with mothers to identify individual factors that may have contributed to the neonatal death. Two in-depth interviews were held with auxiliary nurse midwives (ANMs) and with in-charge of local health institutions each. One in-depth interview was conducted with in-charge of newborn ward in district hospital. Due to limited resources, the number of qualitative data collection was determined to two of each type as at least two may represent the variation in the community. One interview was conducted with the In-charge of the newborn ward of the hospital which was the only one government hospital at the district level. Each FGD required 1 h, while the in-depth interviews and case studies took 45 min to 1 h.

### Definition of variables

Neonatal death was defined as the death of a new born baby during the 28 days of their life. A child who survived the neonatal period was defined as a ‘survivor’.

A number of independent variables were included in the study based on existing literature. Ethnicity was categorized into: (1) advantaged: Upper castes and advantaged Janjati, and (2) disadvantaged: Other Janjati and Dalits; mother’s occupation into: (1) housewife, and (2) service/business/farmer (employed); economic status of family into: (1) poor, and (2) better; education of mother into: (1) no schooling, and (2) primary or higher due to small sample size; age of mothers was recorded as continuous variable and categorized into: (1) 20–35 years, and (2) <20 or >35 years; birth order was recorded as continuous variable and categorized into: (1) 1st, and (2) 2nd or more; number of antenatal care visits into: (1) <4, and (2) ≥4; place of delivery into: (1) health facility, and (2) home/on the way; birth attendants into: (1) skilled birth attendant (doctor, nurse or auxiliary nurse midwife), and (2) non skilled attendants (birth attendant other than those in skilled); post-natal care as: (1) yes if had attended any visits for check up within 42 days after childbirth. Cord cutting was categorized into: (1) clean when either boiled or new blade was used for cord cutting, and (2) unclean (other than used in clean). Low birth weight was defined as birth weight of <2500 g (2.5 kg).

### Data analysis

Quantitative data were entered and analyzed using Statistical Package for Social Sciences (version 16). The association between predictors (socio-demographic factors, antenatal, delivery and postnatal care) and neonatal death was examined using McNemar’s Chi square test at 5 % level of significance. Multivariable backward conditional logistic regression analysis was applied to estimate adjusted odds ratio, along with 95 % confidence interval. For qualitative data, each FGD, in-depth interview and case study were transcribed in Nepali language, and later translated into English. Themes were prepared based on already defined issues included in the guidelines. Narrative analysis was done manually and a summary of each interview and FGD was done.

### Ethics statement

This study was approved by the Nepal Health Research Council. Written permission was also received from the District Public Health Office, Chitwan and informed consent was obtained from all respondents before data collection.

## Results

### Quantitative findings

**Fig. 1:**
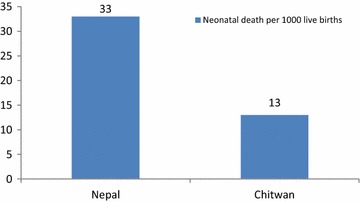
Neonatal mortality rate of Nepal and Chitwan

Figure 1 shows a comparison of the national NMR [10] to that of the district of Chitwan as found in this study using DPHO data collected through the CB-NCP in 2011.

#### Socio-demographic characteristics

Table 1 presents the socio-demographic characteristics of 198 mothers included in this study. About three quarters of mothers included in this study belonged to disadvantaged ethnic groups, three quarters of mothers were in better economic condition and about two in five mothers were only housewives. Two-thirds of mothers reported primary or higher level education, and three quarters of mothers were in the age group 20–35 years. 44 % of the newborns were the first birth.

**Table 1:** Socio-demographic characteristics of the study population

Characteristics	Frequency (N = 198)	Percent
Ethnicity		
Disadvantaged	144	73
Advantaged	54	27
Economic status		
Poorer	50	25
Better	148	75
Occupation		
Employed	120	61
Housewife	78	39
Educational status		
No schooling	68	34
Primary or higher	130	66
Age group of mother		
20–35 years	148	75
<20 or >35 years	50	25
Birth order		
1st	87	44
2nd or more	111	56
Sex of child		
Male	107	54
Female	91	46

#### Maternal health service utilization and newborn care

Majority of respondents (86 %) had ANC checkup, and 64 % had 4 or more ANC checkups. Nearly two-thirds (62 %) of the mothers had delivered in health facility, and 63 % had assistance from a skilled birth attendant during their childbirth. About two-thirds (65 %) of mothers had received postnatal checkup, all of them had cut the cord by clean blade (boiled or new), nothing was applied on umbilicus of 93 % newborns, and drying and wrapping of newborns before placenta delivery was done for 92 % and 91 % of newborns, respectively (Table 2).

**Table 2:** Distribution of maternal health service utilization and newborn care

Characteristics	Frequency (N = 198)	Percent
ANC check up		
No	27	14
Yes	171	86
ANC number		
<4 ANC	72	36
≥4 ANC	126	64
Place of delivery		
Home/on the way	76	38
Health facility	122	62
Birth attendant		
SBA	125	63
Non SBA	73	37
PNC		
No/Do not know	70	35
Yes	128	65
Cord cutting		
Clean	198	100
Unclean	0	0
Applied something on cord		
No	185	93
Yes	13	7
Dried		
No	16	8
Yes	182	92
Wrapping		
No	17	9
Yes	181	91

#### Predictor variables associated with neonatal deaths

Variables showing significant associations with neonatal deaths in McNemar Chi square test have been presented in Table 3. Except birth order and sex of the newborn, all other variables included in the study were significantly associated with the neonatal deaths.

**Table 3:** Association of predictor variables with neonatal deaths

Variables	Outcome of live birth		P value*
	Survivor N (%)	Dead N (%)	
Education			
No schooling	24 (35)	44 (65)	0.006
Primary or higher	75 (58)	55 (42)	
Ethnicity			
Disadvantaged	70 (49)	74 (51)	<0.001
Advantaged	29 (54)	25 (46)	
Occupation			
Employed	57 (48)	63 (52)	0.050
Housewife	42 (54)	36 (46)	
Economic status			
Poorer	22 (44)	28 (56)	<0.001
Better	77 (52)	71 (48)	
Age group			
20–35 years	72 (49)	76 (51)	<0.001
<20 and >35 years	27 (54)	23 (46)	
Birth order			
1st	42 (48.3)	45 (51.7)	0.276
2nd or more	57 (51.4)	54 (48.6)	
Sex of child			
Male	48 (45)	59 (55)	0.505
Female	51 (56)	40 (44)	
Birth weight			
Less than 2.5 kg	6 (15)	35 (85)	<0.001
2.5 kg or more	93 (59)	64 (41)	
ANC check-up			
No	10 (37)	17 (63)	<0.001
Yes	89 (52)	82 (48)	
ANC number			
<4 ANC	30 (42)	42 (58)	0.013
≥4 ANC	69 (55)	57 (45)	
Place of delivery			
Home/On the way	29 (38)	47 (62)	0.042
Health Institution	70 (57)	52 (43)	
Assistant during delivery			
SBA	71 (57)	54 (43)	0.005
Non SBA	28 (38)	45 (62)	
Postnatal care			
No	28 (40)	42 (60)	0.008
Yes	71 (56)	57 (44)	
Applied something on cord			
No	88 (48)	97 (52)	<0.001
Yes	11 (85)	2 (15)	
Drying			
No	2 (13)	14 (87)	<0.001
Yes	97 (53)	85 (47)	
Wrapping			
No	2 (12)	15 (88)	<0.001
Yes	97 (54)	84 (46)	

* P value from McNemar’s Chi square test

Table 4 presents the factors that were significantly associated with neonatal deaths in multivariable regression. After adjusting for other factors in the model, birth weight of newborn, application of something on cord, wrapping of newborn and educational status of mothers remained significant. Newborns with low birth weight were more than 8 times [adjusted odds ratio: 8.49, 95 % CI (3.21–22.47)] more likely to die in neonatal period compared to their counterparts with normal weight. Similarly, applying nothing on cord [adjusted odds ratio: 5.72, 95 % CI (1.01–32.30)] and not wrapping newborn immediately after birth [adjusted odds ratio: 9.54, 95 % CI (2.03–44.73)] were associated with higher likelihood of neonatal deaths. Likewise, newborns born to mothers with no schooling [adjusted odds ratio: 2.09, 95 % CI (1.07–4.11)] were more likely to die in neonatal period compared to newborns of mothers with primary or higher level education.

**Table 4:** Results of multivariable backward conditional logistic regression analysis

Variables	Adjusted odds ratio (95 % CI)	P value
Education		
No schooling	2.09 (1.07–4.11)	0.032
Primary or higher	1	
Birth weight		
Less than 2.5 kg	8.49 (3.21–22.47)	<0.001
2.5 kg or more	1	
Applied something on cord		
No	5.72 (1.01–32.30)	0.048
Yes	1	
Wrapping		
No	9.54 (2.03–44.73)	0.004
Yes	1	

−2 loglikelihood ratio: 222.848; Hosmer-Lemmeshow goodness of fit: 0.347

*CI* Confidence interval

### Qualitative findings

#### Common practices during childbirth

All respondents, along with an ANM of a local birthing center stated that most of the births in Chitwan took place at hospital. In case of home delivery, people called either FCHVs or Traditional Birth Attendants (TBAs) and they used safe delivery kit. Some FCHVs added that deliveries occurred at home because of delay of ambulance coming.

“Most of the deliveries are conducted in health institution and they are cared there. Only very few deliveries occur at home.”—ANM of a primary health care centre

Even in case of hospital delivery, after returning from the hospital, people called TBA for massaging mother and baby with oil, care of newborn, and washing clothes.

“If women deliver at home, they call TBA. Many women who go for institutional delivery also call TBA for massage to mother and infant, keep the newborn warm and wash clothes”.—FCHV

#### Practice of caring neonates

Most common practices of caring neonates were massaging oil to baby, wrapping with warm clothes, bathing after 24 h, and early initiation of breast feeding. They wiped the baby and wrapped in cloth. FCHVs also advised for early initiation of breast feeding and for not using oil and keep baby’s cord dry. In case of hospital delivery, FCHV visited recently delivered mothers on the third day of the delivery.

“Home deliveries are few. If women deliver at home, they call FCHV & FCHV have delivery kit. They use kit, cord cutting is done in an aseptic way. They do not apply anything on cord.”—ANM of a Primary Health Care Centre

“Even in case of home delivery, bathing occurs after 24 h. Cleaning with soft clothes and wrapping in clean clothes is in practice.”—FCHV

#### Delays as causes for neonatal deaths

Two major delays: delays in seeking care and delays in provision of adequate care emerged as cause of neonatal deaths in in-depth interviews and case studies.

“For those who admit late and are with complication it is difficult to handle them. Many people don’t recognize the danger signs and become late. Some of them have problems for transportation and money to come to hospital on time.”—In-charge of newborn ward of hospital

While reaching hospital and accessing services are important issues, negligence by doctors and nurses and the lack of knowledge of danger signs of neonatal deaths among mothers were cited as the reasons of neonatal death.

“Before one day of incident, I had little watery discharge. So, I went to hospital and I had to be admitted. In the morning, at about 7 am doctor came for checkup. He said that baby had eaten own faeces and had to be operated soon. Operation was done, my daughter was 3.2 kilograms in weight. She was seriously ill, therefore, she was taken to ICU. Next day, she died there. I did not have chance to see her. One thing I want to tell, doctor and nurse neglected patient. I was admitted one day before the event, they should have checked me and operated one day before. But doctor rather told me that the condition was not bad on the first day. It could have been known from Video X-ray. But they had not advised for that”.—A case study of a mother

“I had normal delivery in Bharatpur hospital. Baby was girl with 3 kg weight. She could not suck milk and did not urinate. Sister (nurse) told me to milk in spoon and feed the child. During delivery, doctor & nurse both helped. But they did not come later to check me. Next day, doctor came & discharged me. Till then, child had not started sucking milk. Child passed stool but did not urinate. Sister told me child urinated during defecation but I did not notice that. I was discharged & returned home. Child started sucking but did not suck properly, used to sleep a lot, started breathing rapidly on next day. I did not know that these are danger signs, so I waited for two days. I did not guess this will happen. At night the child was serious. We went to primary health care centre but it was closed. Then we went to a private clinic. In the medical my sister was told that the baby was very serious, was bluish and there was no chance of survival. We returned home and baby died after some hours.”—A case study of a mother

#### Improvements in neonatal survival

As presented earlier, a community based newborn care program is on-going in the district. Key informants indicated that awareness through FCHV, communication through cell phone, and rise in institutional delivery had contributed to the reduction of neonatal death.

“It [neonatal death] is decreasing rapidly. In past many neonates used to die but nowadays we can hardly hear that, one or two in a year.”—ANM of a sub health post

“Increased awareness among people, 24-h delivery services in rural health facility and increased institutional delivery has contributed to decrease neonatal mortality.”—Staff nurse of neonatal ward of public hospital

“After [implementation of] CB-NCP, FCHVs visit mother [for newborn care and counseling of mothers on proper care of newborn]. Nowadays, communication is also easy through mobile phone.”—ANM of a sub health post.

#### How neonatal deaths can be reduced further

Most of the participants said that 24 h delivery service from each peripheral health facility and increasing awareness especially of daughters-in-law and mothers-in-law could reduce the neonatal death in the community.

“Increasing the number of birthing centres [to increase facility deliveries], utilizing FCHV to their optimum level; and managing proper and timely available transport system can decrease neonatal mortality. Health education in community, school, especially to adolescents can be useful.”—In-charge of a sub health post

Nurse of the neonatal ward of the district public hospital indicated that facilities to address complicated cases at the hospital as well as timely referral of such patients from the lower level health institution were required to save newborns after they reach to health facility.

“There must be well equipped district hospital to manage complicated cases referred from the peripheral health facility. Service from hospital should be effective & efficient. We are lacking neonatal ICU. It can prevent many neonates life. There should be timely referral by peripheral staff nurse.”—Staff nurse of neonatal ward of hospital

## Discussion

The study aimed at investigating the factors associated with neonatal deaths in Chitwan district of Central Nepal. Significantly lower neonatal mortality rate was reported in Chitwan compared to the national statistics (Fig. 1). A number of reasons can be attributable to such lower neonatal mortality. The district is relatively accessible to health services which has two large medical universities and a tertiary level hospital to provide services for severely ill neonates [11]. Well built transportation system except for few hilly villages can also facilitate easy access to health services in times of need. In addition, the district is one of first districts implementing community based programs on ARI and Diarrhoea. All of the health workers and FCHVs have been trained to provide community based newborn care to increase child survival. FCHVs encourage mothers for facility delivery [6]. They visited mother and newborn within first 3 days to support and teach mothers on optimum newborn care such as cord care, thermal care, and kangaroo mother care in case of low birth weight newborns.

The important findings of this study are the associations of low birth weight, application of something on cord, wrapping of newborn and educational status of mother with neonatal mortality. The study found that there was 2 times higher likelihood of neonatal death when mothers had no schooling compared to the neonates with mothers having primary or higher education. Consistent results were found in a study in Bangladesh where the risk of neonatal death was found 5.6 folds higher among mothers having no formal education than mothers with higher education [13]. The current study found that low birth weight increased the risk of neonatal deaths by more than 8 times compared to those having normal or more birth weight. This is consistent with findings from Zimbabwe 4.67 (95 % CI 3.92–5.57) and Iran 7.68 (95 % CI 1.49–39.55) [14, 15]. Similarly, a Nigerain study also reported low birth weights having 4.7 times more risk to die during neonatal period compared to newborns with birth weight 2.5 kg or more [16]. The low birth weight newborns are likely to suffer more from hypothermia, infection, and poor immunological function which increase risk of neonatal deaths. PNC should include extra care for low birth weight babies with regard to promotion of healthy behaviours and identification of danger signs [17].

The results from our quantitative findings indicate that essential newborn care (cord care and thermal care) were the major factors associated with higher likelihood of newborn deaths. Qualitative findings indeed also provided the similar suggestion regarding the delays in care. Delay in care seeking has been well established as one of the three major reasons for maternal deaths [5]. A further focus is needed to educate mothers to seek for the treatment and care of newborn during illness.

Case studies revealed that some of the mothers perceived that their newborns were not provided with adequate care and attention by the health workers even in hospital. This finding highlights the third delay according to the three delay model [5]. This is an alarming finding in Nepal where the newborn death is a major challenge [12] and care seeking is poor. Such perception of poor care in hospital may discourage mothers to seek treatment from hospitals [18]. Such findings reiterate that those delays need to be the major focus while implementing any neonatal survival programs in Nepal. Additionally, providing complete information on the procedure that the newborns are getting in the intensive care unit and indoor care is essential. This approach requires a better communication skill of health care workers which the current medical education system is lacking.

The findings of this study are important for newborn survival program in Nepal and there are two areas of policy implication from our study findings: (1) prevention of low birth weight births, and (2) improved essential newborn care. A recent study has shown that attending recommended four antenatal care visit and consuming iron supplementation during pregnancy was protective against low birth weight in Nepal [19]. Therefore, FCHVs and health workers should encourage mothers for such services and ensure adequate nutrition to pregnant women. Further, provision of newborn care should be provided using the opportunity of recently implemented newborn care programs. Future studies might show the benefits of these programs, however, our study did support the fact that it will be useful to have community-based newborn care programs in Nepal. However, due to resource intensiveness and changing dynamics of the population mobility in Nepal, such programs should use on-going operational research to revise along the way. There are a number of limitations that should be considered while interpreting the findings of this study. Some important predictor variables such as complication in newborn, birth interval and quality of care at health facility have not been assessed quantitatively although the qualitative study explored some information. Small sample size is another major limitation of this study. Nevertheless, current findings do suggest important implications for newborn survival in Nepal.

## Conclusion

Low birth weight, not wrapping newborns, applying nothing on cord and no schooling of mothers were significantly associated with higher likelihood of neonatal mortality. This has implications for behavioural and health service related interventions in preventing low birth weight and promoting newborn care practices. Further, empowerment of women through education would also contribute in the survival of newborns.
